# Concentric Needle Jitter in 97 Myasthenia Gravis Patients

**DOI:** 10.3389/fneur.2020.600680

**Published:** 2020-11-13

**Authors:** João Aris Kouyoumdjian, Gabriel Pina Paiva, Erik Stålberg

**Affiliations:** ^1^Laboratório Investigação Neuromuscular, Faculdade Estadual Medicina São José do Rio Preto, São Paulo, Brazil; ^2^Department of Clinical Neurophysiology, Institute of Neurosciences, Uppsala University, Uppsala, Sweden

**Keywords:** jitter, single-fiber electromyography, myasthenia gravis, concentric needle electrode, electrical activation

## Abstract

**Objectives:** To estimate the jitter parameters (single-fiber electromyography) in myasthenia gravis patients mostly by electrical activation in *Frontalis, Orbicularis Oculi*, and *Extensor Digitorum* muscles using a concentric needle electrode.

**Methods:** Between 2009 and 2019, a total of 97 myasthenia gravis patients, 52 male, and mean age 54 years were included.

**Results:** Any abnormal jitter parameter in individual muscles was 90.5% (*Frontalis*), 88.5% (*Orbicularis Oculi*), and 86.6% (*Extensor Digitorum*). Any jitter parameter combining *Orbicularis Oculi* and *Frontalis* muscle was abnormal in 100% for the ocular, and in 92.9% for the generalized myasthenia gravis. The most abnormal muscle was *Orbicularis Oculi* for the generalized, and *Frontalis* for the ocular myasthenia gravis. The decrement was abnormal in 78.4%, 85.9% for the generalized, and 25% for the ocular myasthenia gravis. The mean jitter ranged from 14.2 to 86 μs (mean 33.3 μs) for the ocular myasthenia gravis and from 14.4 to 220.4 μs (mean 66.3 μs) for the generalized myasthenia gravis. The antibody titers tested positive in 86.6%, 91.8% for the generalized, and 50% for the ocular myasthenia gravis. Thymectomy was done in 48.5%, thymoma was found in 19.6%, and myasthenic crisis occurred by 21.6%.

**Conclusion:** The jitter parameters achieved a 100% abnormality in ocular myasthenia gravis if both the *Orbicularis Oculi* and *Frontalis* muscles were tested. There was a high jitter abnormality in generalized myasthenia gravis cases with one muscle tested, with about a 2% increase in sensitivity when a second is added. Concentric needle electrode jitter had high sensitivity similar to the single fiber electrode (93.8%), followed by antibody titers (86.6%), and abnormal decrement (78.4%).

## Introduction

The neuromuscular transmission is compromised in several diseases, like myasthenia gravis (MG). It can be evaluated by the single-fiber electromyography (SFEMG), a technique developed in the early 1960s by Erik Stålberg and Jan Ekstedt in Sweden (1–4). The test measures the neuromuscular jitter parameters, reported as numerical jitter values and frequency of impulse blocking. The SFEMG jitter represents the variation in time interval between pairs of single fiber action potentials (SFAPs) in the voluntarily activated technique or in the time measured between stimulation pulse and SFAPs in the stimulated technique. With high jitter values, the neuromuscular transmission is so disturbed, that occasional or complete impulse blocking occurs. The jitter parameters and the associated impulse blocking are the most sensitive electrodiagnostic signs of the neuromuscular junction (NMJ) dysfunction.

Myasthenia gravis is an autoimmune disorder affecting NMJ, usually associated with antibodies to the acetylcholine receptor (AChR) in about 85%. In 10–20% of patients with generalized MG (GMG) and about 50% of patients with ocular MG (OMG), there are no detectable antibodies to the AChRs (5). Among the seronegative MG cases, antibodies to muscle-specific kinase (anti-MuSK) can be found in about 40% or low-density lipoprotein receptor-related protein 4 (anti-LRP4) in ~9%. The thymus gland has a significant role in MG, and a thymectomy is a therapeutic option for those patients. Either thymic hyperplasia (majority) or thymoma (10–15%) could be found.

The sensitivity of SFEMG is about 88% in OMG and 95–100% in GMG (6). Similarly, in a systematic review, Benatar (7) confirmed a high specificity of SFEMG for the diagnosis of GMG. For OMG, the sensitivity ranged from as low as 62% (7) to as high as 100% (8, 9). Mercelis and Merckaert (10) found a specificity of 97% and a sensitivity of 80% for OMG in stimulated-SFEMG for *Orbicularis Oculi* (OO). It should be stressed that increased jitter is not equal to myasthenic disorder but is also seen in other conditions, particularly reinnervation (4). Increase jitter is a sign of disturbed neuromuscular transmission.

Disposable concentric needle electrodes (CNE) is currently used for jitter measurement (5, 11), due to the increasing concern for the transmission of infections. Some papers have presented normative data and the diagnostic value of the test in MG (5, 9, 11–21). Farrugia et al. (22) found no difference in mean jitter values for *Extensor Digitorum* (ED) and OO muscles in 24 MG patients using both single fiber electrode (SFE) and CNE. Papathanasiou and Zamba-Papanicolaou (23) found no significant difference between mean jitter measured by disposable or reusable SFE in 18 MG patients in the OO muscle stimulation technique. To improve recording selectivity and successfully use a CNE for jitter measurement, the low-frequency filter should typically be raised from 500 Hz to 1 or 2 kHz to suppress the activity from distant muscle fibers. A filter setting with a 1 kHz high pass filter, rather than higher, has been suggested for optimal quality (24). This setting seems to balance the desired effect of low-frequency suppression with a reasonably preserved original signal shape and an acceptable signal-to-noise ratio. Usually, the high frequency (low pass) filter is 10 kHz. In recordings with noisy low amplitude signals, a filter of 3 kHz is recommended, particularly when the jitter is measured with the peak method. Different amplitude criteria, such as 50, 100, or 200 μV have been used in literature, but the most important is that acceptable ASFAPs should be clear solitary spikes with a fast-rising slope to a well-defined negative peak with a constant shape in consecutive discharges (25). In our study, the signal should have an amplitude exceeding 100 μV to be accepted. As the signals obtained with CNE recording do not always represent a single fiber action potential, but rather a summation of many, the term jitter recording with CNE from Apparent Single Fiber Action Potential (ASFAPs) (12), is preferable, rather than SFEMG with CNE. In our previous reports (26, 27), we have used electrical stimulation for jitter measurements in 20 and 42 MG patients. This study aimed to evaluate a larger cohort of MG patients (including those previously reported using electrical (mostly) and voluntary activation CNE jitter in ED, OO, and *Frontalis* (FR) muscles.

## Materials and Methods

### Patients

Between August 2009 and September 2018, we retrospectively studied hundred-five patients, highly suspected of having MG. They were referred for CNE jitter measurements. Patients to be eligible for being included had to present at least one of the following; fluctuating weakness, unequivocal clinical response to pyridostigmine or other anticholinesterase drugs, and at least one of the following; positive acetylcholine receptor antibody (AbAChR) titer (>0.40 nmol/L), positive muscle-specific tyrosine kinase antibody (AbMuSK) titer (≥0.02 nmol/L), or decrement of at least 10% on slow (2–3 Hz) repetitive nerve stimulation (RNS) studies. The first of their jitter studies being reported. The severity of disease was determined clinically according to the Myasthenia Gravis Foundation of America (MGFA) clinical classification from I, ocular weakness, to II-V, generalized weakness (28), and defined clinically from “*the worst through disease*,” and “*the best after treatment*” at the last visit. Worst MGFA was considered for the OMG or GMC distinction. Concentric needle electrode jitter recordings and antibody measurements, either to AbAChR or MuSK, were studied the same day while RNS may have been performed at another time up to 21 years separation in time.

### Repetitive Nerve Stimulation (RNS)

**Figure 1:**
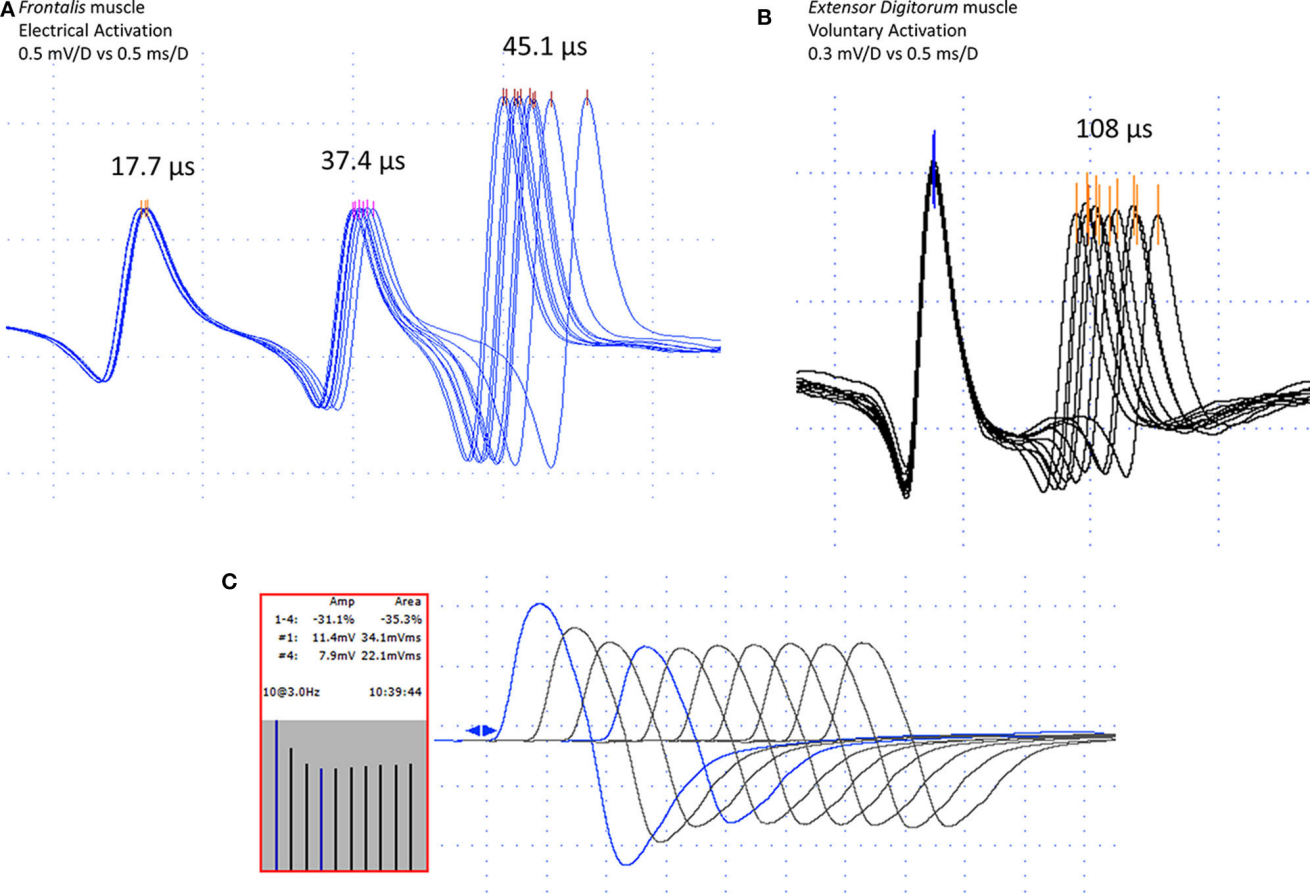
Jitter recording (peak triggering) with concentric needle electrode after electrical **(A)** and voluntary **(B)** activation in myasthenia gravis cases. In **(A)**, one normal (17.7 μs), and two abnormal jitters (37.4 and 45.1 μs). In **(B)**, a pair of abnormal jitter (108 μs). Repetitive nerve stimulation (3 Hz), in the median nerve to the *Abductor Pollicis Brevis* muscle, showing typical decrement, fourth to the first response, 31.1% for the amplitude, and 35.3% for the area **(C)**. Note high-quality acquisition for both, the repetitive nerve stimulation, and the jitter measurement with fast-rising phase spikes without notches or shoulders and well-defined peak without shape changes at consecutive discharges.

Most recordings were done in the Portable Keypoint or KeypointNet electromyograph (Medtronic Skovlunde, Denmark) and some in the *Natus*™ *UltraPro (USA)* machine. A few cases came from other services, and the RNS was done in different machine trades. Repetitive nerve stimulation was done in all patients, and the most pronounced and reliable quality recording decrement was considered anytime since the MG beginning. Several nerve-muscle settings, either distal, proximal, or facial, were done. Routine rules for RNS were followed. The distal limb temperature was kept warm (>32°C). The patients on cholinesterase inhibitors (mostly pyridostigmine) were asked to withhold the medication 24 h before the test if medically not contraindicated. The limb tested was adequately immobilized. Adhesive superficial electrodes were used. A supramaximal stimulation was delivered, about 10–20% above the intensity level needed for a maximal response. There was no rigorous protocol, but most of the cases had been tested for at least two muscles, distal limbs, and either proximal or facial unless the first test showed an unequivocal abnormal decrement. The most commonly nerve-muscles studied were the median nerve to the *Abductor Pollicis Brevis* (APB) muscle (Figure 1), the ulnar nerve to the ADM muscle, the spinal accessory nerve to the *Trapezius* muscle, the facial nerve to the OO muscle, the facial nerve to the *Nasalis* muscle, the facial nerve to the *Orbicularis Oris* muscle, and the radial nerve to the *Anconeus* muscle. All RNS tests were done at rest and with low-frequency stimulation (2–3 Hz) with 7 or 10 stimuli. A decremental response higher than 10% of the forth to the first amplitude or area (the highest decrement value) response was considered abnormal. In all cases, the compound muscle action potentials had an amplitude within normal limits. In 25 cases, the RNS studies were done at rest and repeated 2, 3, and 4 min after 60 s of strong voluntary activation. Maximal decrement was considered.

### Jitter Recording

All jitter measurements were performed on the Natus™ Keypoint-Net (USA, former Medtronic Skovlunde, Denmark) or Natus™ UltraPro (USA) machines with in-built software developed explicitly for the SFEMG test. The measurement of jitter parameters was done using disposable concentric needle electrodes (CNE) of two brands: Ambu® Neuroline Concentric, 25 × 0.30 mm (30G), recording area 0.02 mm^2^ and, Natus™ Dantec® DCN Disposable Concentric Needle Electrode, 25 × 0.30 mm (30G), recording area 0.02 mm^2^. Both electrodes are routinely used and approved by the Brazilian Agency for Health Surveillance (ANVISA).

The tests were done according to the recent guideline reported for the jitter studies (4). We used either peak or amplitude level detection algorithm for time measurements. The recordings were performed using a CNE with a diameter of 0.30 mm and a recording area of 0.019 mm^2^ (the smallest or “*facial needle*” CNE from Medtronic/Alpine BioMed, Denmark). The first author (JAK) performed all jitter measurements, and the last author (ES) revised, when necessary, the digital recordings. Cholinesterase inhibitors may mask abnormal jitter when the abnormality of neuromuscular transmission is mild (4). For this reason, the patients asked to withhold the medication at least 12 h before the test if medically not contraindicated, like the routines for RNS.

The ED was tested 67 times, 60 with electrical and seven with voluntary activation. The FR was tested 74 times, 72 with electrical and two with voluntary activation. The OO was tested 29 times, 23 with electrical and six with voluntary activation.

Recordings were made from a minimum of three electrode positions with at least two skin CNE insertions with radial advancement into the muscle. The jitter value was calculated as the mean consecutive differences (MCD) for each pair of potentials (voluntarily activation), or each spike (electrical activation), using ideally 100 pair/spikes (we accepted 50 in some circumstances).

The MCD variation was measured by the “*amplitude level*,” i.e., by the temporal variation of the ascending lines of depolarization of the ASFAPs, or by “*the peak*,” i.e., by the temporal variation of the mathematically defined peaks of the ASFAPs. Acceptance of potentials was based on the criteria established in the literature summarized in Sanders et al. (25), as follows: the depolarization ascending lines must be parallel without notches of shoulders, the potentials should have a constant shape on consecutive discharges, best seen on superimposition; the APs peak must be regular; the APs amplitude should be the same, but small variations are tolerated (Figure 1). The skin temperature was maintained at more than 30°C. Reference jitter parameters for CNE were taken from the multicenter reference study (21). The mean jitter after voluntary activation is abnormal if the value was higher than 30 μs (ED muscle), 31 μs (OO muscle), and 28 μs (FR muscle). The mean jitter after electrical activation is abnormal if the value is higher than 24 μs (ED muscle), 27 μs (OO muscle), and 21 μs (FR muscle). The individual jitter values are abnormal after voluntary activation if they are higher than 43 μs (ED muscle), 45 μs (OO muscle), and 38 μs (FR muscle). The individual jitter values are abnormal after electrical activation if they are higher than 35 μs (ED muscle), 36 μs (OO muscle), and 28 μs (FR muscle). The jitter analysis was considered abnormal if: (1) the mean jitter was above the reference value described above; (2) more than 2 or (3) jitter values for voluntary and stimulation methods, respectively were above the reference value, outliers.

### Percutaneous Electrical Activation Technique

The most common electrical activation technique used here was performed with a bar electrode to stimulate the temporalis (FR muscle) or the zygomatic branch (OO muscle) of the facial nerve percutaneously. We find the appropriate location for the bar fixation by adjusting the stimulus intensity to produce a slightly visible muscle twitch. In general, this could be achieved at about 5–7 mA. Stimulation parameters were 10 Hz frequency (the most closely to the physiological one) using rectangular pulses of 0.10 ms duration. The recording position and stimulus intensity were adjusted to give a minimum number of spikes in the response. The most important here is to assure that each spike accepted for analysis had a supraliminal stimulation. If a spike showed increased jitter, the intensity was raised slightly to check for correct stimulus intensity. When the stimulus intensity was raised from subliminal to adequate, the spike latency decreased somewhat, the jitter decreased, the impulse blocking disappeared. Frequently some more spikes came out, but these must be skipped or undergo the test for adequate stimulation.

### Intramuscular Microaxonal Electrical Activation Technique

The intramuscular microaxonal stimulation technique was less commonly used than the surface stimulation and was mainly chosen for studies of the ED muscle. Disposable monopolar needle electrode [Ambu® Monopolar Neuroline, × 0.36 mm (28G), AMBU A/S (DK-2750 Ballerup)], 25 mm, was introduced into the muscle near the motor point (active); another similar electrode (reference) was introduced 2–3 cm in any direction, to the insertion site of the active electrode, following standards already described (4, 14, 29). The stimulation frequency was settled to 10 Hz; the stimulation intensity (mA), in the form of square pulses of 0.04 ms, was adjusted to produce small visible local muscle twitching, usually achieved with intensity <2 mA. The same disposable CNE described previously was inserted in the area of visible muscle twitching, and adjusted for the registration of the ASFAPs. Thirty MCDs of 30 different motor endplates were measured. Care was taken to exclude jitter values <5 μs (direct muscle fiber stimulation), the “*axon-reflex*,” and F-waves (30, 31). The other electrophysiological parameters were the same as those already described in the previous technique.

### Voluntary Activation

The CNE was introduced into the chosen muscle, and after minimal maintained voluntary contraction, the ASFAPs were recorded. After slight electrode movements, a pair of ASFAPs was recorded, with both potentials belonging to the same motor unit. The trigger was usually set to the highest amplitude spike. We excluded the ASFAP pairs without clear separation between them (>300 μs), the records with <50 ASFAP pairs, the ASFAP pairs with interpotential interval (IPI) >4 ms to avoid the effect of velocity recovery function (VRF), or when the shape criteria were not fulfilled. The mean value of the sorted differences (MSD) was also calculated, which expresses the consecutive differences according to the potential pairs' frequency of discharge. When the inter-potential interval (IPI) variation is higher than 4 ms and not constant, there may be interference from the VRF; in a few situations when it occurs we use the MSD if the MCD/MSD ratio is higher than 1.25, or with recent software, the smaller of the two parameters is chosen automatically. The other electrophysiological parameters were the same as those already described in the previous technique.

### Statistics

Descriptive statistics calculated the mean jitter (MCD) values of the 97 MG patients and all other parameters: mean and standard deviation for normal or Gaussian distribution or median/percentage for non-normal distribution. Anderson-Darling normality test was used. The Student's *t*-test made the comparison of parametric variables with a significance of 0.05. The comparison of non-parametric variables was made by the Mann-Whitney non-parametric test (*U*-test) to verify the medians' equality. Correlation of the mean jitter values (MCD) with variables was made with a linear model, calculating the R-square to show the correlation power of jitter in the regression line.

## Results

### Clinical and Demographical Findings

From the 105 cases selected, we excluded 8, leaving 97 cases for analysis. The exclusion was based on the full constellation of normal jitter parameters, no abnormal decrement, no antibody increased titers either to AChR or MuSK, no or just a disputable fluctuating weakness, and no response to anticholinesterase inhibitor drugs or prednisone for at least a year. With all tests still negative for MG for 1 year, alternate diagnoses were considered.

The majority of the cases (74/97 or 76.3%) considered themselves free of symptoms for daily activities. Overall, 94 (96.9%) were taking daily pyridostigmine, 53 (54.6%), regular or alternated prednisone, and 26 (26.8%), azathioprine. One case was in remission (medicine-free). The most frequent combination was pyridostigmine plus prednisone (65 cases, 67%), and pyridostigmine, prednisone plus azathioprine (24 cases, 24.7%). Just one patient used the combination pyridostigmine plus azathioprine. No circumstances went to death due to the MG itself. Three cases died from other reasons; aortic aneurysm, acute myocardial infarction, and heart failure, one case each. Thymectomy was performed on 47 (48.5%) patients with thymoma found in 40.4% (19.6% from the total). Myasthenic crisis occurred in 21 cases (21.6%) without fatalities (Table 1).

**Table 1:** Some variable parameters from all, generalized, and ocular myasthenia gravis cases.

	**All**	**Generalized**	**Ocular**
*n*	97	85	12
Male	53.6%	51.8%	66.7%
Age	54 (17.9)	53.4 (16.9)	55.7 (24.6)
Age of debut	43.3 (19.6)	42.3 (18.5)	50.8 (25)
Time of symptoms (months)	61.9 (1–330)	62.6 (1–330)	56.6 (2–228)
Thymectomy	48.5%	54.1%	8.3%
Thymoma	19.6%	22.4%	0%
Myasthenic crisis	21.6%	24.7%	0%
Pyridostigmine	98.9%	96.5%	100%
Prednisone	70.5%	70.6%	41.7%
Azathioprine	26.8%	30.6%	0%
AbAChR abnormal	84.5%	89.4%	50%
AbAChR mean value (nmol/L)	14.1	15.9	1.84
AbMuSK abnormal	2.1%	2.4%	0%
AbAChR or AbMuSK abnormal	86.6%	91.8%	50%
Striated muscle antibody abnormal	22.7%	24.7%	8.3%
Cases with abnormal decrement	78.4%	85.9%	25%
Mean percentage of decrement	25.9%	28.7%	5.9%
Any abnormal jitter parameter	93.8%	92.9%^*^	100%^**^
Some impulse blocking	19.3%	20.9%	5.8%

* *One muscle tested = 91.3%, and two muscles tested = 93.5%*.

** *In 5/10 cases, just one of two muscles tested was abnormal*.

*AbAChR, acetylcholine receptor antibody; AbMuSK, muscle-specific kinase antibody (SD)*.

The worst MGFA was considered for the OMG or GMC distinction. Ocular MG was defined for 12 cases (12.4%), meaning that patients had maintained just ocular symptoms (eyelid drop and diplopia) for at least 2 years (in one case, 20 months). Generalized MG was defined for 85 cases (87.6%), meaning that patients had extensive weakness outside the ocular muscle symptoms. Some of the variables are shown in Table 1. Ten men and nine women constituted the group with a thymoma (19 cases). For those, the mean start age was 39 ± 13.2 years (17–68), the AbAChR titer was positive in 94.7% (mean 19.9 ± 26.6 nmol/L, 0.3–85), the antibody titer to striated muscle tissue was positive in 42.1%, the decrement was abnormal in 84.2%, and any jitter parameter was abnormal in 94.7%.

### Antibodies

The mean abnormal AbAChR titer was 14.14 ± 21.05 nmol/L (range, 0.07–100). The AbAChR was abnormal in 84.5% and antiMuSKAb in 2.1%. The two MuSK positive patients were AbAChR negative (Table 1). In eight cases with abnormal titer for antibody to striated muscle tissue, a thymoma was found, one had thymus hyperplasia, and one had normal thymus.

### Repetitive Nerve Stimulation

Abnormal decrement was found in 76/97 patients (78.4%) at any time of the disease, meaning that the RNS and the jitter analysis plus antibodies could be more than 10 years apart. In total, 258 tests were done with a mean of 2.7 per patient (1–8). We excluded 11 tests done in the same muscle, both sides, leaving 247 tests for analysis. In cases where multiple nerve-muscles were tested, we chose the one with the highest abnormal decrement. The most pronounced decrement was found in facial muscles in 48.5% of the cases, hand muscles in 37.1%, and proximal muscles in 14.4% (Table 2). There was no defined RNS protocol due to the retrospective data analysis.

**Table 2:** Mean worst decrement from the tested muscles in 97 myasthenia gravis cases.

**Muscle**	***n***	**Percentage (%)**	**Mean decrement (%)**	**Minimal (%)**	**Maximum (%)**
*Orbicularis Oculi*	26	26.8	18.6	0.0	66.6
*Abductor Digiti Minimi*	18	18.6	26.8	4.8	50.0
*Abductor Pollicis Brevis*	18	18.6	28.9	4.0	60.5
*Orbicularis Oris*	11	11.3	30.1	0.0	59.0
*Trapezius*	10	10.3	33.0	14.0	61.0
*Nasalis*	10	10.3	25.0	2.9	49.0
*Anconeu*s	4	4.1	29.6	6.7	58.0

Amplitude and area parameters showed a similar degree of change in stimulation. When only one of them was abnormal, the study was considered abnormal (this occurred in one case). In a comparison between facial and the distal group, eight cases were only abnormal in facial muscles, and in two instances, just the distal muscles were abnormal. All abnormal proximal muscles share the abnormality with the facial muscles. Despite the increase of the decrement after 2–4 min of 60 s of maximal effort in eight cases (25 nerve-muscles), in only 1, the values went from normal (<10%) to abnormal (more than 10%).

### Jitter Parameters

**Figure 2:**
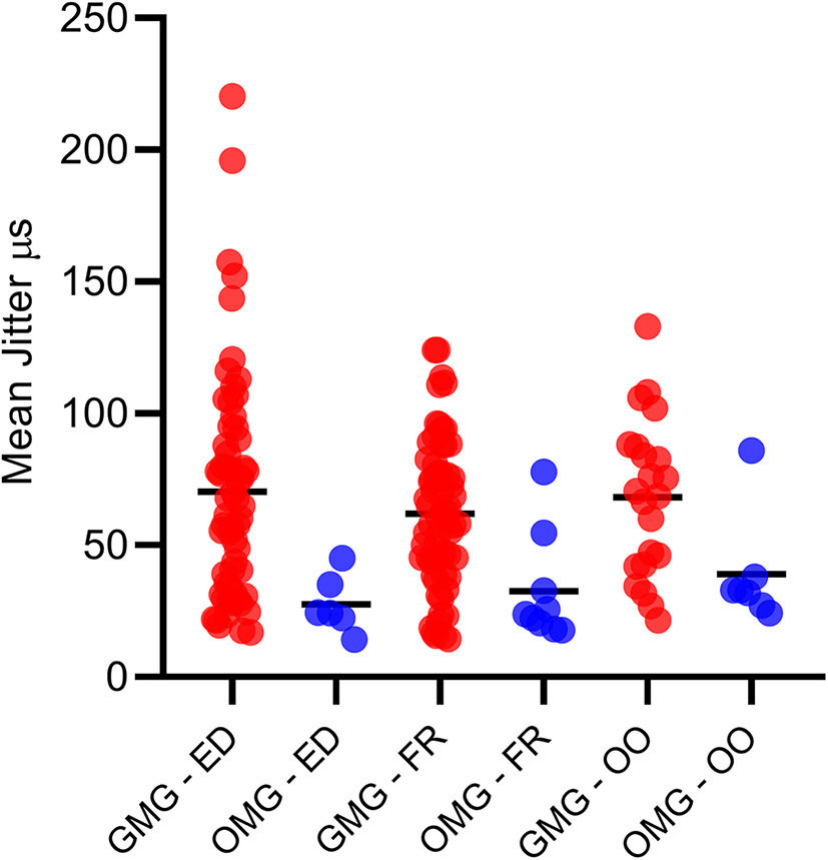
All mean jitter values for the generalized (GMG) and ocular myasthenia gravis (OMG) in *Extensor Digitorum, Frontalis*, and *Orbicularis Oculi* muscles. Mean = continuous dash.

A total of 170 muscles were tested. Overall, 91/97 MG patients (93.8%) had at least one muscle and one abnormal jitter parameter. Overall, 79/85 GMC patients (92.9%) had at least one muscle and one abnormal jitter parameter. Overall, 12/12 OMG patients (100%) had at least one muscle and one abnormal jitter parameter. Just one muscle was tested in 25 patients, two muscles in 71 patients, and three muscles in one patient. The jitter parameters in all tested muscles for the GMG and OMG are displayed in Figure 2. In most cases, 20 pairs (voluntary activation), and 30 ASFAPs (electrical activation) was reached. Overall, 150/171 (87.7%) muscles had at least one abnormal jitter parameter, either the mean jitter above the limit or more than 10% of abnormal individual jitter values. When two muscles were tested (71 cases), the abnormality was found in just one muscle in 9 cases. Regardless the muscle studied and activation technique, the mean jitter ranged from 14.2 to 86 μs (mean 33.3 μs) for the OMG and from 14.4 to 220.4 μs (mean 66.3 μs) in GMG.

**Figure 3:**
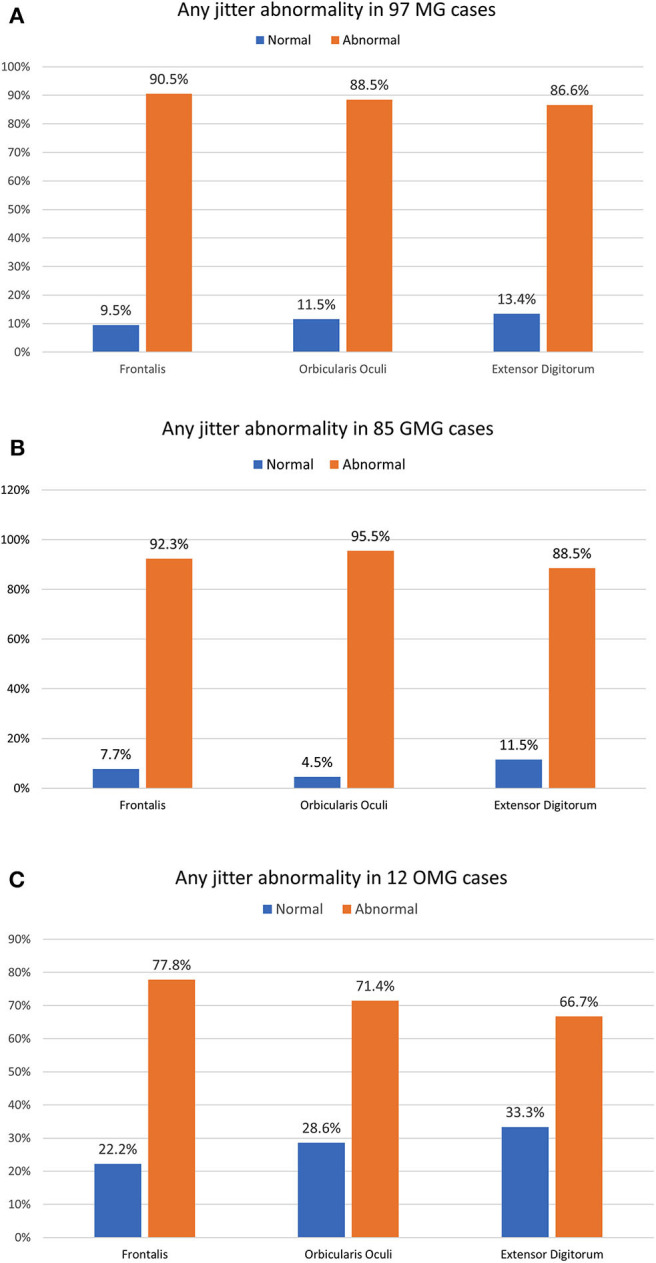
The percentage of any jitter abnormality parameter in three individually muscles for 97 myasthenia gravis (MG) cases **(A)**, 85 generalized MG cases **(B)**, and 12 ocular MG cases **(C)**. The OMG jitter abnormality only reached the 100% abnormality with the combination of the *Frontalis* and the *Orbicularis Oculi* muscle. The GMG jitter abnormality was very high, even in just one muscle studied.

For the OMG, neither OO (71.4% abnormal) nor FR (77.8% abnormal) individually could provide a 100% abnormality. The combination of the mean jitter measurement for the OO and the FR muscles added 5/12 abnormal cases and reached 100% abnormality. For the GMC group (85 cases), the combination of two tested muscles helped for detecting neuromuscular transmission dysfunction in just two additional cases, and the percentage of cases with abnormal jitter for any jitter parameter went from 90.6 to our final number of 92.9%. The overall sensitivity for all cases and GMC and OMG forms are shown in Figure 3.

In patients with GMG and OMG, we found spikes with an impulse blocking in 20.9 and 5.8%, respectively, either ED, FR, or OO muscles. Overall, impulse blocking was found in 19.3%. Impulse blocking in MG cases after therapy (similar to this study) was found in 5.2% (ED muscle) and 20.6% (FR muscle) for ocular and 9.9–38.3% (ED muscle) and 20.6–47.6% (FR muscle) for generalized forms (4). In our previous studies (26, 27) with 20 and 42 MG cases we found 22.4 and 21.5% for the ocular form, and 34.6 and 25.5%, respectively, more than we found here, and probably due to the more severe cases in those reports. In voluntarily activated jitter, the percentage of recordings with blocking may be higher compared to stimulation jitter due to the block in one of two fibers will be counted as a blocking pair.

### Parameters in Ocular vs. Generalized Cases

Despite the small cohort, a comparison between OMG (worst MGFA class I, 12 patients) and GMG (worst MGFA class II–V, 84 patients) was done for some parameters and is shown in Table 1.

## Discussion

We retrospectively reviewed a larger MG cohort than our previous report (26, 27) measuring jitter parameters with CNE. The cohort was heterogeneous as expected for retrospective and observational studies. Some patients selected for jitter analysis and AbAChR or AbMuSK titers determination included already confirmed MG cases under treatment for months or years from diagnosis. Some are new MG cases. As long as the fatigue was restricted to the ocular muscles for 2 years (20 months in one case), the group was assigned as OMG and represented 12 (12.4%). The remaining 85 cases (87.6%) were defined as GMG, with fatigue extending outside the ocular muscles.

This observational, consecutive, and retrospective survey was designed to study the general feasibility of CNE jitter analysis in MG. The results do not represent the techniques' sensitivity and specificity. All three of the most studied muscles were used for the activation, ED, OO, and FR. Abnormal jitter was found in 93.8% for any, regardless of MG type. This percentage was more than 90%, and 90.5% found in our previous reports (26, 27) may be due to more rigorous criteria for MG and close to 92% described by others (4).

Our results showed the requirement to test two muscles (OO and FR were tested in this material) for the OMG in most since the isolated muscle jitter results may not fully reflect the abnormality, whereas the combination FR and OO gave 100%.

For the GMG, any abnormal jitter parameter was found in 92.9%. Our results showed the requirement of two muscle tests for the GMG would be unnecessary in most since the isolated muscle jitter results are abnormal in a high percentage. If the first muscle is normal, a second muscle should be tested, increasing the sensitivity by just 2%. This may indicate a more even distribution of abnormalities among muscle in GMA than in OMG.

The analysis of the six patients without abnormality of any jitter parameters showed 4 cases with previous abnormal decrement obtained 9–21 years earlier, and three cases with abnormal AbAChR titers, confirming a neuromuscular transmission dysfunction in all cases at some time. This unexpected finding of the discrepancy between jitter and RNS may be explained by the delay between the tests. In the two examples with striking previous abnormal decrement and negative abnormal AbAChR titers, a congenital myasthenic syndrome should be suspected. In our previous studies (4, 26, 27) similar results were obtained.

Overall, the decrement was found abnormal in 78.4% of all MG cases, 85.7% for the GMG, and 25% for the OMG. In general, these figures are similar to many of the published studies. Exceptions occur depending on type and severity of MG, type of test protocol and muscle chosen. Openness to unexpected findings and flexibility in testing protocols are necessary. In eight cases, an abnormal decrement was found only in the facial muscles, and in two cases was found only in the distal muscles. This finding is unusual, usually proximal, or facial muscles have a larger decrement than distal muscles. These findings stress the importance of distal studies in all cases when proximal or facial muscles have normal decrement. Also, a systematic bilateral exploration of at least a facial muscle (OO or *Nasalis*), the *Trapezius*, and the *Anconeus* could increase in 33% the test sensitivity (32). Amandusson et al. (33), found the *Deltoideus* muscle as the most sensitive, regardless of MG subtype, when the abnormal limit was set at 5%.

Stålberg et al. (4) found an abnormal decrement in hand or proximal muscles (*Deltoideus* more abnormal than *Trapezius*) in 75% of patients with GMG, the same as here. In OMG and OMG, RNS was reported abnormal in 10–48 and 48.5%, respectively (4, 34). The OO muscle was the most frequent abnormal muscle (26.8%), as already described (35). The relatively high proportion of the abnormal decrement in the hand muscles probably reflects the RNS test protocol, starting in this segment, and when abnormal, no other muscle is tested. Only a few reports described our unusual finding of abnormal decrement only in the hand muscles in 2 cases (2.1%). Accordinly, Stålberg (36), studying 164 MG patients, could not find a single case where the hand muscles have shown a decrement, and the proximal muscles have been normal at the same time. Later studies (4) have shown a few occasions of abnormality only in hand muscles. Niks et al. (37), studying the RNS for both *Nasalis* and ADM muscles, found 3/25 MG cases in whom only the ADM muscle presented abnormal decrement. Zambelis et al. (38), studying 115 MG patients, found one case (1.1%) with abnormal decrement only in the ADM with predominant limb-axial weakness. Recently, Lee and Li (39), reported four patients (4.2%), a double from our results, abnormal decrement was observed only in the APB muscle, while the *Trapezius* and the facial muscles were normal. They claimed that the distal weakness in MG is frequently overridden. In a study of 70 seropositive MG patients, weakness in the distal upper extremity exceeded that seen proximally in 37% (40). There is no consensus about the utility of the area instead of the amplitude of the compound muscle action potential to measure decrement (35).

In the present study, abnormal percentage of outliers were a little higher or equal to abnormal mean jitter, similar to our previous studies (26, 27). The mean jitter is usually a more reliable parameter of abnormality than the number of outliers in the voluntary activation technique because the jitter recording is obtained with two motor endplates (4). If one motor endplate has low normal jitter, and the other barely above normal values for individual neuromuscular junctions, the combined jitter of the two would be normal.

Due to the distinguishing CNE recording area difference to the SFE, a separated normative data had been collected (21). Some reports which have compared jitter values from SFEMG and CNE in healthy controls and patients with MG reported a good correlation between the results with the same accuracy for the neuromuscular transmission defect detection (5, 10, 11). After some previous reports on stimulation jitter analysis with CNE in healthy subjects (14, 15, 19, 41) and patients with MG (17, 26), we realize that in the ocular muscles the individual single fiber signals may be shorter and more separated in time from each other compared to ED. This technique can be applied in pediatric patients (42) after a collection of reference values for younger age groups, but here are ASFAP difficult to obtain. Large limb muscles like ED are less recommended for stimulation studies.

We are aware that our data have some good points and also some limitations. There was a large cohort, at least for CNE jitter studies; the data contemplated both activation techniques, electrical and voluntary for the jitter measurements; and the main points of clinical correlation was achieved (demographic data, ocular or generalized, thymectomy, thymoma, myasthenic crisis, and medications in use). Our primary purpose here was related to the CNE jitter analysis). The first limitation is that the study was observational and retrospective. We have different protocols for some electrophysiological data as RNS and sequence of examining muscles for the jitter measurements. Besides, the activation technique was not the same for all patients. The second limitation is that we could not routinely test for the congenital myasthenic syndrome or congenital myopathies when there was no response to MG therapy. It occurred in three cases with negative antibodies titers. A third limitation is the RNS and jitter studies may have been performed many years apart, and we may have a completely different clinical situation on two occasions. However, these limitations do not influence the main results and conclusions.

In conclusion, the present study confirms that CNE can be reliably used for the jitter analysis and impulse blocking for a neuromuscular transmission dysfunction suspicion as MG. For the GMG, we found a high rate of abnormality for any jitter parameters, regardless of one or more muscles were studied. For the OMG, we found a very high percentage of abnormality for any jitter parameters (100%) only using combined muscles, OO, and FR in the same exam. Therefore, in OMG, a second facial muscle should be studied if the first showed normal results. These findings were similar to what has been reported in a more extensive series with SFE. Technically, CNE jitter measurement was much more easily obtained, less painful, and less time consuming for the facial muscles than for the ED. We found the combined distal and ocular RNS studies was the most useful for having a decremental response.

## Data Availability Statement

The raw data supporting the conclusions of this article will be made available by the authors, without undue reservation.

## Ethics Statement

The studies involving human participants were reviewed and approved by Ethics committee of the Faculdade Estadual de Medicina de São José do Rio Preto (FAMERP), Av. Faria Lima 5416, 15090-000, São Paulo, Brazil, where the SFEMG test was performed. The patients/participants provided their written informed consent to participate in this study.

## Author Contributions

JK and ES contributed to the conceptual project design, statistical analysis, data interpretation, and manuscript draft. JK did all SFEMG tests. GP contributed to the data collection and management. All authors contributed to the manuscript's critical review and approval.

## Conflict of Interest

The authors declare that the research was conducted in the absence of any commercial or financial relationships that could be construed as a potential conflict of interest.

## Abbreviations

AbAChR: anti-acetylcholine receptor antibody
AbMuSK: anti-muscle-specific tyrosine kinase antibody
AChR: acetylcholine receptor
ASFAP: apparent single fiber potential
CNE: concentric needle electrode
ED: *Extensor Digitorum* muscle
FR: *Frontalis* muscle
LRP4: low-density lipoprotein receptor-related protein 4
MCD: mean consecutive difference
MG: myasthenia gravis
MGFA: Myasthenia Gravis Foundation of America
MuSK: muscle-specific tyrosine kinase
NMJ: neuromuscular junction
OO: *Orbicularis OO* muscle
RNS: repetitive nerve stimulation
SFE: single fiber electrode
SFEMG: single-fiber electromyography.

## References

[1] Ekstedt J Human single muscle fiber action potentials. Acta Neurol Scand. (1964) 61:1–96. 14150641

[2] Stålberg E Trontelj JV. Single Fiber Electromyography. Studies in Healthy and Diseased Muscle. 2nd ed. New York, NY: Raven Press (1994).

[3] Sanders DB Stålberg E. AAEM minimonograph #25: single-fiber electromyography. Muscle Nerve. (1996) 19:1069–83. 10.1002/(SICI)1097-4598(199609)19:9<1069::AID-MUS1>3.0.CO;2-Y 8761262

[4] Stålberg EV Trontelj JV Sanders DB Single Fiber EMG. 3rd ed Fiskebäckskil: Edshagen Publishing House (2010).

[5] Benatar M Hammad M Doss-Riney H. Concentric-needle single-fiber electromyography for the diagnosis of myasthenia gravis. Muscle Nerve. (2006) 34:163–8. 10.1002/mus.20568 16642500

[6] Sanders DB Howard JF. AAEE minimonograph #25: single-fiber electromyography in myasthenia gravis. Muscle Nerve. (1986) 9:809–19. 10.1002/mus.880090904 3785290

[7] Benatar M. A systematic review of diagnostic studies in myasthenia gravis. Neuromuscul Disord. (2006) 16:459–67. 10.1016/j.nmd.2006.05.006 16793269

[8] Padua L Stålberg E Lo Monoco M Evoli A Batocchi A Tonali P. SFEMG in ocular myasthenia gravis diagnosis. Clin Neurophysiol. (2000) 111:1203–7. 10.1016/S1388-2457(00)00307-2 10880794

[9] Machado FCN Kouyoumdjian JA Marchiori PE. Diagnostic accuracy of concentric needle jitter in myasthenia: prospective study. Muscle Nerve. (2016) 55:190–4. 10.1002/mus.25229 27348087

[10] Mercelis R Merckaert V. Diagnostic utility of stimulated single-fiber electromyography of the *Orbicularis Oculi* muscle in patients with suspected ocular myasthenia. Muscle Nerve. (2011) 43:168–70. 10.1002/mus.21853 21254079

[11] Sarrigiannis PG Kennett RP Read S Farrugia ME. Single-fiber EMG with a concentric needle electrode: validation in myasthenia gravis. Muscle Nerve. (2006) 33:61–5. 10.1002/mus.20435 16175626

[12] Ertaş M Baslo MB Yildiz N Yazici J Öge AE. Concentric needle electrode for neuromuscular jitter analysis. Muscle Nerve. (2000) 23:715–9. 10.1002/(SICI)1097-4598(200005)23:5<715::AID-MUS8>3.0.CO;2-V 10797394

[13] Kouyoumdjian JA Stålberg E. Concentric needle single fiber electromyography: normative jitter values on voluntary activated *Extensor Digitorum*. Arq Neuropsiquiatr. (2007) 65:446–9. 10.1590/S0004-282X2007000300016 17665013

[14] Kouyoumdjian JA Stålberg E. Concentric needle single fiber electromyography: comparative jitter on voluntary-activated and stimulated *Extensor Digitorum*. Clin Neurophysiol. (2008) 119:1614–8. 10.1016/j.clinph.2008.03.008 18455474

[15] Kouyoumdjian JA Stålberg E. Reference jitter values for concentric needle electrodes in voluntarily activated *Extensor Digitorum* and *Orbicularis Oculi* muscles. Muscle Nerve. (2008) 37:694–9. 10.1002/mus.21043 18506720

[16] Kokubun N Sonoo M Imai T Arimura Y Kuwabara S Komori T Reference values for voluntary and stimulated single-fiber EMG using concentric needle electrodes: a multicentre prospective study. Clin Neurophysiol. (2012) 123:613–20. 10.1016/j.clinph.2011.07.044 21889397

[17] Delgado-Delos Santos MMS Rosales RL Stimulated single fiber electromyography using a concentric needle electrode among normal filipino subjects. Clin Neurophysiol. (2012) 123:e51 10.1016/j.clinph.2011.11.201

[18] Lozano AMR Corredor FO Single fiber electromyography with a concentric needle electrode and surface stimulus in the *Orbicularis Oculi* muscle. Clin Neurophysiol. (2012) 123:e17–68. 10.1016/j.clinph.2011.11.209

[19] Kouyoumdjian JA Stålberg EV Concentric needle jitter in stimulated frontalis in 20 healthy subjects. Muscle Nerve. (2012) 45:276–8. 10.1002/mus.22306 22246886

[20] Sanders DB Measuring jitter with concentric needle electrodes. Muscle Nerve. (2013) 47:317–8. 10.1002/mus.23709 23203567

[21] Stålberg E Sanders DB Ali S Cooray G Leonardis L Löseth S. Reference values for jitter recorded by concentric needle electrodes in healthy controls: a multicenter study. Muscle Nerve. (2016) 53:351–62. 10.1002/mus.24750 26112058

[22] Farrugia ME Weir AI Cleary M Cooper S Metclafe R Mallik A. Concentric and single fiber needle electrodes yield comparable jitter results in myasthenia gravis. Muscle Nerve. (2009) 39:579–85. 10.1002/mus.21151 19260051

[23] Papathanasiou ES Zamba-Papanicolaou E. A comparison between disposable and reusable single fiber needle electrodes in relation to stimulated single fiber studies. Clin Neurophysiol. (2012) 123:1437–9. 10.1016/j.clinph.2011.10.046 22119663

[24] Stålberg E Sanders DB. Jitter recordings with concentric needle electrodes. Muscle Nerve. (2009) 40:331–9. 10.1002/mus.21424 19705424

[25] Sanders DB Arimura K Cui L Ertaş M Farrugia ME Gilchrist J Guidelines for single fiber EMG. Clin Neurophysiol. (2019) 130:1417–39. 10.1016/j.clinph.2019.04.005 31080019

[26] Kouyoumdjian JA Fanani ACS Stålberg EV. Concentric needle jitter on stimulated frontalis and extensor digitorum in 20 myasthenia gravis patients. Muscle Nerve. (2011) 44:912–8. 10.1002/mus.22203 22102462

[27] Kouyoumdjian JA Stålberg EV. Stimulated jitter with concentric needle in 42 myasthenia gravis patients. Arq Neuropsiquiatr. (2013) 71:237–43. 10.1590/0004-282X20130008 23588285

[28] Jaretzki A III Barohn RJ Ernstoff RM Kaminski HJ Keesey JC Penn AS. Myasthenia gravis. Recommendations for clinical research standards. Task force of the medical scientific advisory board of the myasthenia gravis foundation of America. Neurology. (2000) 55:16–23. 10.1212/WNL.55.1.16 10891897

[29] Trontelj JV Mihelin M Fernandez JM Stålberg E. Axonal stimulation for endplate jitter studies. J Neurol Neurosurg Psychiatry. (1986) 49:677–85. 10.1136/jnnp.49.6.677 3016197 PMC1028851

[30] Stålberg E Sanders DB Kouyoumdjian JA. Pitfalls and errors in measuring jitter. Clin Neurophysiol. (2017) 128:2233–41. 10.1016/j.clinph.2017.09.001 29017138

[31] Stålberg E Kouyoumdjian JA Paiva G Yanaze LL Sanders DB. Problems in comparing jitter values obtained with voluntary activation and electrical stimulation. J Neuromuscul Dis. (2018) 5:225–30. 10.3233/JND-170289 29614693

[32] Ali HB Salort-Campana E Grapperon AM Gallard J Franques J Sevy A. New strategy for improving the diagnostic sensitivity of repetitive nerve stimulation in myasthenia gravis. Muscle Nerve. (2017) 55:532–8. 10.1002/mus.25374 27511866

[33] Amandusson A Elf K Grindlund ME Punga AR. Diagnostic utility of repetitive nerve stimulation in a large cohort of patients with myasthenia gravis. J Clin Neurophysiol. (2017) 34:400–7. 10.1097/WNP.0000000000000398 28872522

[34] Meriggioli MN Sanders DB Myasthenia gravis: diagnosis. Semin Neurol. (2004) 24:31–9. 10.1055/s-2004-829594 15229790

[35] Chiou-Tan FY Gilchrist JM Repetitive nerve stimulation and single-fiber electromyography in the evaluation of patients with suspected myasthenia gravis or Lambert–Eaton myasthenic syndrome: review of recent literature. Muscle Nerve. (2015) 53:455–62. 10.1002/mus.24745 26109387

[36] Stålberg E. Clinical electrophysiology in myasthenia gravis. J Neurol Neurosurg Psychiatry. (1980) 43:622–33. 10.1136/jnnp.43.7.622 6249895 PMC490629

[37] Niks EH Badrising UA Verschuuren JJ van Dijk JG. Decremental response of the nasalis and hypothenar muscles in myasthenia gravis. Muscle Nerve. (2003) 28:236–8. 10.1002/mus.10411 12872330

[38] Zambelis T Kokotis P Karandreas N. Repetitive nerve stimulation of facial and hypothenar muscles: relative sensitivity in different myasthenia gravis subgroups. Eur Neurol. (2011) 65:203–7. 10.1159/000324915 21412008

[39] Lee TH Li Y. Consideration of repetitive nerve stimulation of the median nerve in patients being evaluated for myasthenia gravis. Muscle Nerve. (2019) 60:658–61. 10.1002/mus.26713 31531870

[40] Oztürk A Deymeer F Serdaroglu P Parman Y Ozdemir C. Distribution of extremity muscle weakness in myasthenia gravis: sparing of tibialis anterior muscle. Acta Myol. (2003) 22:58–60. 14959565

[41] Kouyoumdjian JA Stålberg E. Concentric needle jitter on stimulated *Orbicularis Oculi* in 50 healthy subjects. Clin Neurophysiol. (2011) 122:617–22. 10.1016/j.clinph.2010.07.012 20708431

[42] Tidswell T Pitt MC. A new analytical method to diagnose congenital myasthenia with stimulated single-fiber electromyography. Muscle Nerve. (2007) 35:107–10. 10.1002/mus.20637 16941657

